# Supplementary material for: Overestimation of school-based deworming coverage resulting from school-based reporting
Source: PLoS Negl Trop Dis. 2023 Apr 10;17(4):e0010401. doi: 10.1371/journal.pntd.0010401 (PMC10118084; doi:10.1371/journal.pntd.0010401)
Supplement: S1 Table — Cluster-level statistics of the proportion of SAC attending the school that is geographically closest to their home, the total number of students, and the number of unique schools reported as being attended by students in each cluster, replicated for each of the three study sites. (DOCX) [file pntd.0010401.s001.docx]

**Supplementary Table 1. Cluster-Level School Age Children Statistics, by Study Site**

Cluster-level statistics of the proportion of SAC attending the school that is geographically closest to their home, the total number of students, and the number of unique schools reported as being attended by students in each cluster, replicated for each of the three study sites.

| Cluster ID | Benin | | | India | | | Malawi | | |
| --- | --- | --- | --- | --- | --- | --- | --- | --- | --- |
|  | Proportion Attending Nearest School | Total Students (n) | Schools (n) | Proportion Attending Nearest School | Total Students (n) | Schools (n) | Proportion Attending Nearest School | Total Students (n) | Schools (n) |
| 1 | 0.18259 | 471 | 16 | 0.203883 | 206 | 9 | 0.393983 | 698 | 6 |
| 2 | 0.471572 | 299 | 8 | 0.191781 | 219 | 11 | 0.793497 | 1138 | 13 |
| 3 | 0 | 94 | 8 | 0.521472 | 163 | 9 | 0.357771 | 682 | 12 |
| 4 | 0.245098 | 204 | 10 | 0.173267 | 202 | 13 | 0.46888 | 723 | 9 |
| 5 | 0.654788 | 449 | 6 | 0.0625 | 192 | 15 | 0.316344 | 569 | 12 |
| 6 | 0.426667 | 375 | 18 | 0.147147 | 333 | 15 | 0.723353 | 835 | 11 |
| 7 | 0.580986 | 568 | 12 | 0.341709 | 199 | 12 | 0.997337 | 751 | 3 |
| 8 | 0.523691 | 401 | 11 | 0.246305 | 203 | 11 | 0.972185 | 755 | 11 |
| 9 | 0.207824 | 409 | 13 | 0.026042 | 192 | 10 | 0.997503 | 801 | 3 |
| 10 | 0.536797 | 231 | 15 | 0.127358 | 212 | 12 | 0.927294 | 839 | 8 |
| 11 | 0.40146 | 137 | 18 | 0.184 | 250 | 16 | 0.918685 | 578 | 4 |
| 12 | 0.132275 | 189 | 20 | 0.22807 | 171 | 16 | 0.82459 | 610 | 7 |
| 13 | 0.329317 | 249 | 10 | 0.136792 | 212 | 14 | 0.948454 | 679 | 4 |
| 14 | 0.365385 | 208 | 18 | 0.113971 | 272 | 15 | 0.98188 | 883 | 9 |
| 15 | 0.387234 | 235 | 16 | 0.103806 | 289 | 15 | 0.989418 | 756 | 6 |
| 16 | 0.119231 | 260 | 20 | 0.44375 | 160 | 17 | 0.777879 | 1094 | 8 |
| 17 | 0.045918 | 196 | 28 | 0.409396 | 298 | 17 | 0.006579 | 608 | 7 |
| 18 | 0.2875 | 80 | 17 | 0.504348 | 230 | 16 | 0.982044 | 724 | 9 |
| 19 | 0.220721 | 222 | 11 | 0.394886 | 352 | 10 | 0.870886 | 790 | 6 |
| 20 | 0.169591 | 171 | 17 | 0.225225 | 222 | 11 | 0.990442 | 837 | 6 |
| 21 | 0.113402 | 194 | 29 | 0.066667 | 285 | 9 | 0.752146 | 932 | 8 |
| 22 | 0.092308 | 65 | 20 | 0.086705 | 173 | 9 | 0.948598 | 856 | 8 |
| 23 | 0.61008 | 377 | 15 | 0.302439 | 205 | 7 | 0.908075 | 805 | 8 |
| 24 | 0.07619 | 105 | 14 | 0.277108 | 249 | 9 | 0.973006 | 815 | 3 |
| 25 | 0.3 | 170 | 20 | 0.271429 | 140 | 7 | 0.861789 | 492 | 5 |
| 26 | 0.102041 | 245 | 13 | 0.366071 | 112 | 4 | 0.988304 | 513 | 5 |
| 27 | 0.079096 | 177 | 11 | 0.302158 | 139 | 5 | 0.158627 | 1078 | 16 |
| 28 | 0.118421 | 76 | 11 | 0.242188 | 128 | 6 | 0.231106 | 1601 | 21 |
| 29 | 0.212389 | 113 | 19 | 0.339869 | 153 | 5 | 0.968796 | 673 | 5 |
| 30 | 0.145161 | 124 | 22 | 0.215517 | 116 | 7 | 0.995716 | 1167 | 5 |
| 31 | 0.051282 | 117 | 18 | 0.191083 | 157 | 6 | 0.750554 | 902 | 6 |
| 32 | 0.006329 | 158 | 16 | 0.119403 | 201 | 7 | 0.512518 | 679 | 5 |
| 33 | 0.142857 | 196 | 14 | 0.151703 | 323 | 23 | 0.814902 | 1275 | 9 |
| 34 | 0.024845 | 161 | 23 | 0.122378 | 286 | 19 | 0.728333 | 1200 | 6 |
| 35 | 0.295082 | 183 | 17 | 0.206557 | 305 | 21 | 0.883573 | 1254 | 10 |
| 36 | 0.25 | 232 | 8 | 0.043659 | 481 | 17 | 0.983577 | 1096 | 7 |
| 37 | 0.415225 | 289 | 5 | 0.174912 | 566 | 31 | 0.89523 | 1174 | 12 |
| 38 | 0.973373 | 338 | 8 | 0.302491 | 281 | 22 | 0.447806 | 661 | 14 |
| 39 | 0.610932 | 311 | 12 | 0.128205 | 429 | 24 | 0.805617 | 1353 | 13 |
| 40 | 0.402344 | 256 | 9 | 0.364548 | 299 | 24 | 0.95443 | 790 | 3 |
